# Brain Networks for Integrative Rhythm Formation

**DOI:** 10.1371/journal.pone.0002312

**Published:** 2008-05-28

**Authors:** Michael H. Thaut, Martina Demartin, Jerome N. Sanes

**Affiliations:** 1 Center for Biomedical Research in Music, Colorado State University, Fort Collins, Colorado, United States of America; 2 Molecular, Cellular, and Integrative Neuroscience Program, Colorado State University, Fort Collins, Colorado, United States of America; 3 Institute of Experimental Medicine, National Research Council, Rome, Italy; 4 Department of Neuroscience, Alpert Medical School of Brown University, Providence, Rhode Island, United States of America; 5 Laboratory of Functional Neuroimaging, Foundation Santa Lucia, Istituto di Ricovero e Cura a Carattere Scientifico (IRCCS), Rome, Italy; University of Birmingham, United Kingdom

## Abstract

**Background:**

Performance of externally paced rhythmic movements requires brain and behavioral integration of sensory stimuli with motor commands. The underlying brain mechanisms to elaborate beat-synchronized rhythm and polyrhythms that musicians readily perform may differ. Given known roles in perceiving time and repetitive movements, we hypothesized that basal ganglia and cerebellar structures would have greater activation for polyrhythms than for on-the-beat rhythms.

**Methodology/Principal Findings:**

Using functional MRI methods, we investigated brain networks for performing rhythmic movements paced by auditory cues. Musically trained participants performed rhythmic movements at 2 and 3 Hz either at a 1∶1 on-the-beat or with a 3∶2 or a 2∶3 stimulus-movement structure. Due to their prior musical experience, participants performed the 3∶2 or 2∶3 rhythmic movements automatically. Both the isorhythmic 1∶1 and the polyrhythmic 3∶2 or 2∶3 movements yielded the expected activation in contralateral primary motor cortex and related motor areas and ipsilateral cerebellum. Direct comparison of functional MRI signals obtained during 3∶2 or 2∶3 and on-the-beat rhythms indicated activation differences bilaterally in the supplementary motor area, ipsilaterally in the supramarginal gyrus and caudate-putamen and contralaterally in the cerebellum.

**Conclusions/Significance:**

The activated brain areas suggest the existence of an interconnected brain network specific for complex sensory-motor rhythmic integration that might have specificity for elaboration of musical abilities.

## Introduction

Considerable evidence suggests that the basal ganglia, cerebellum, and neocortex contribute to temporal encoding and perception related to movement production [1]–[4], though each region's role requires additional elucidation. Studying rhythmic movements synchronized to sensory stimuli has proven useful in uncovering behavioral and neural aspects of action timing [5]–[15]. Converging evidence shows that pulse-salient models underlie rhythm formation; these models require synchronization of rhythmic events into felt pulse patterns [16], [17]; these patterns refer to the presence of a temporally equally spaced sequence of auditory events that serve as perceptual reference points to hear sound durations and patterns [18]. The fundamental role of rhythm in many sensory-motor tasks suggests the existence of brain circuits mediating rhythm formation [19]–[22]. However, the connection between behavioral aspects of pulse-salient models of rhythm formation and their neural implementation requires further elaboration to enable greater understanding of brain representations for rhythmic manual performance required not only for common everyday actions but also for specialized tasks, such as performing with musical instruments.

One limitation to understand brain substrates of integrative rhythm formation is that current functional neuroimaging work often investigates rhythm perception and production separately (but see, [23]). Among other structures, listening to a rhythm involves left inferior parietal and prefrontal and bilateral cerebellar circuits [24]–[26]. Rhythm production primarily activates the primary motor (M1), primary somatic sensory (S1), and premotor (PMA) cortices, supplementary motor area (SMA), and lateral cerebellar hemisphere, perhaps for controlling motor timing needed for rhythm elaboration (e.g., [27]–[31]). By contrast, little work has attempted to differentiate brain circuits involved in listening to pacing sounds that can produce different rhythms (but see, [23], [32]).

In this study, we investigated brain representations related to performing 1∶1 stimulus-driven rhythms and hemiola polyrhythmic structures (here 2∶3 and 3∶2), aiming to understand regional specialization for producing rhythms commonly used in musical production. We selected hemiola rhythms since they represent an exceedingly complex time integration task for rhythm formation within the musical domain. Hemiola refers to musical structures in which two beats become replaced by three beats or vice-versa. These structures give the effect of a shift between a double (triple) and a triple (double) meter. The differential computational demands of the two timing tasks could potentially distinguish brain networks using isorhythmicity or polyrhythmicity to produce stimulus-driven movements. Based on prior work [33], [34], we predicted that both experimental conditions would activate the basal ganglia. Furthermore, we predicted that enhanced cerebellar activation would occur only in the hemiola, polyrhythmic condition for which complex neural computations become required to undertake grouping, sequencing and asymmetric mapping of different time intervals into a single rhythmically synchronized pattern. This outcome would agree with recent suggestions about a broader role of the cerebellum in temporal organization fundamental to information processing in many cognitive, sensory, and motor functions, but not explicit timing regarding actual chronometric functions, such as in duration coding. Lastly, we predicted that cortical networks involving frontal-parietal circuits and insular and opercular regions would become activated similarly for both pacing conditions with the latter two possibly involved in sensory-to-motor projections specific to auditory-motor synchronization.

## Materials and Methods

### Participants and Tasks

Nine male and three female young adults (20–36 yr, 26.1±1.8 mean±SEM) without history of neurological disorders volunteered for the study; all 12 individuals participated in the MRI component and seven individuals took part in a subsequent behavioral component of the study. All participants had musical abilities significantly more advanced than the general population and were in professional or semiprofessional employment positions in locally based orchestras and other concert ensembles. Their expertise covered a wide range of orchestral instruments in winds, strings, brass, and percussion. Their instrumental practice averaged ∼1 hr each day. They all reported the need for the capability of executing hemiola polyrhythms regularly during their musical education as well as their current rehearsal and concert duties. Participants were included in the study only if they could perform a 3∶2 and 2∶3 hemiola polyrhythm (see below for description). We note that those musically trained individuals who can perform such a rhythm do so “categorically”; that is, they either have capability to perform the hemiola polyrhythm or they do not. Hemiola rhythms are an integral part of all compositional and improvisational languages in all musical cultures [35], [36], and, in Western music, they have been commonly practiced since the middle ages [37]. They belong in the categorical performance repertoire of all professional musicians, as used in our study [38]–[40]. As noted, our participants were selected on evidence (an initial subjective performance test) that they could perform hemiola rhythms. Ten participants were right-handed according to a revised Edinburgh Inventory [41], while two were ambidextrous. Institutional authorities of Foundation Santa Lucia IRCCS reviewed and approved the project as ethically suitable for human experimentation, and participants provided written informed consent and received modest monetary compensation for undergoing the procedures. Before the imaging component of this study, we subjectively assessed the temporal accuracy of each participant's rhythmic performance that was then later confirmed by time analysis of performance recordings (see below for quantitative assessment procedures). The subjective assessment entailed careful observation of the performance and an interview. Due to their self-knowledge, these musically trained individuals knew *a priori* whether they could perform hemiola polyrhythms; nevertheless, we undertook the initial subjective and subsequent objective assessment for verification. All the procedures related to contact with human volunteers occurred at Foundation Santa Lucia IRCCS, Rome, Italy.

**Figure 1:**
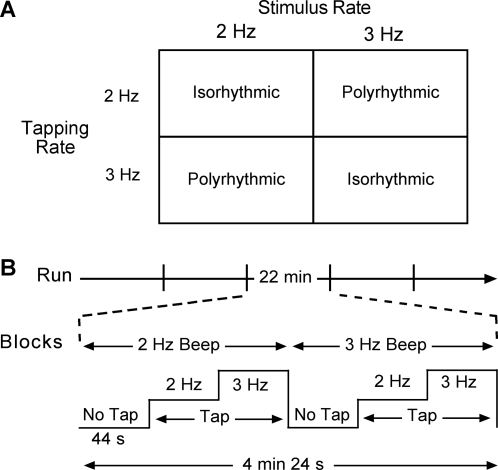
Experimental design and timing for the sensory-motor conditions. A. Experimental design indicating the factorial relationship between the pacing auditory stimulus and the actual tapping. Additional details in text. B. Experimental implementation. The 22 min experiment was divided into five equal blocks, each comprised of six conditions (one condition, “No Tap” was repeated twice). Repeating 2 Hz sounds occurred in the first half of each block and 3 Hz sounds occurred in the second half of a block. Participants first listened to the 2 Hz sounds without moving, then tapped isorhythmically and then polyrhythmically to the 2 Hz beeps. The beeping frequency then changed to 3 Hz and participants first listened, followed by polyrhythmic and then isorhythmic tapping.

With their eyes closed, participants listened to either 2 Hz or 3 Hz beeps (system beep of a Macintosh computer, Apple Computer Corp., Cupertino, CA) through headphones and performed right-handed index finger tapping movements paced in a 1∶1 tapping pattern to beat synchronously with the auditory rhythm or asynchronously in hemiola patterns (2 taps vs. 3 beats or 3 taps vs. 2 beats) to the beat synchrony. Each of the 40 s blocks included one of four isorhythmic or polyrhythmic movements, to yield a total of four combinations of auditory stimuli and tapping: 2 Hz movements in response to 2 Hz (isorhythmic) or 3 Hz (polyrhythmic) beeps; and 3 Hz movements in response to 2 Hz (polyrhythmic) or 3 Hz (isorhythmic) beeps (Fig. 1*A*). For the isorhythmic paced tasks, participants tapped on the beat; for the polyrhythmic paced tasks, participants tapped faster or slower than the pacing stimulus. The required tapping rate relative to the acoustic pacing stimulus was instructed verbally by Italian word equivalents to “same”, “quicker” or “slower”. Before the MRI component of this study, participants received training in the sensory-motor tasks to ensure adequate performance, and during the practice and the actual experiment, participants tapped the right index finger against the right thigh. In the MRI experiment, participants performed five blocks using each auditory stimulus—tapping rate combination for a total of 20 sensory-motor tasks that were interspersed (see below) with no-movement runs; the no-movement runs were verbally instructed by the command “no movement” (see Fig. 1*B*).

The ability to perform hemiola movements was confirmed after the MRI scanning in a sub-set of the 12 participants (7/12), using time analysis of rhythmic performance records (see Results for more details). Due to the categorical nature of their ability to perform the polyrhythm [39], we have no expectation that the participants' performance changed between the time of the MRI session and the behavioral assessment. Participants individually sat in front of a table, donned headphones, and tapped a hand-held electronic stylus against an electronically sensitive plate while listening to the periodic pacing beeps superimposed upon the noise of a previously recorded functional MRI run; these procedures represented a reasonable facsimile of the sensory conditions of the MR environment. The time of contact of the stylus with the plate was measured with 1 ms precision. We used the same task timing as that in the functional MRI blocks (Fig. 1*B*, above for details), but without the no-movement condition. However, in the behavioral experiment, participants performed two task blocks with each auditory stimulus—tapping rate combination. Since the participants had the ability to perform the polyrhythmic, hemiola movements as a categorical skill, the absence of behavioral data from five participants and a delay between the MRI and behavioral segments of the work would not seem to mitigate the MRI (see Discussion and [42]).

### Magnetic resonance imaging

A Vision Magnetom MR system (Siemens Medical Solutions, Erlangen, Germany) operating at 1.5T and equipped for echo-planar imaging acquired the anatomical and functional MR images. A quadrature volume head coil was used for radio frequency transmission and reception. Each participant's head was approximately centered in the standing magnetic field of the MR system once within the MR bore. Head movement was minimized by mild restraint and cushioning.

After positioning a participant in the MR system and performing a shimming procedure to minimize inhomogeneities in the standing magnetic field, we acquired a three-dimensional T1 weighted scout image to aid slice positioning for subsequent acquisition of functional MR images. We then acquired a T1 weighted volumetric image set (1 mm isotropic voxels, 160 sagittal slices, Siemens multiplanar rapid acquisition gradient echo sequence, TR = 11.4 msec, TE = 4.4 msec) for later use to overlay functional MR images. Functional MR images were acquired in a transverse plane roughly parallel to the bi-commissural line using gradient-echo methods sensitive to deoxyhemoglobin concentration [43]. Forty 3 mm thick slices were acquired to encompass the brain using an ascending interleaved excitation order (TR = 3 sec, TE = 40 msec, 64×64 image matrix, 3×3 mm in plane pixel size, 27 mm^3^ voxel size, flip angle = 90°, TR = 4,000 msec, no inter-slice gap).

We used a block design during a single, uninterrupted 22 min functional MR imaging run (Fig. 1*B*), functionally divided into five equal 4 min 24 s blocks. In each block, the 2 and 3 Hz beeps became paired with isorhythmic and polyrhythmic tapping, and participants also listened passively to each of the beeping frequencies. We fixed the task order across participants in each block to optimize performance as follows: 2 Hz beeps with no movement, 2 Hz isorhythmic tapping, 2 Hz polyrhythmic tapping (2 Hz beep, 3 Hz movement; 3 Hz beeps with no movement, 3 Hz isorhythmic tapping, 3 Hz polyrhythmic tapping (3 Hz beep, 2 Hz movement). As noted, each block was repeated five times for a total of 30 stimulus—response tasks. (Pilot experiments indicated that counterbalancing of the stimulus and movement rate combinations severely degraded performance.) The type of task was instructed verbally (see above).

Eleven functional MR volumes were acquired for each 44 s sub-block; an initial 4 s instruction period, following by a 40 s periods of no movement or tapping. Thus, across the entire experimental run 55 volumes became acquired for each task. Note that there were two no-movement conditions, each having 55 volumes of data.

### Behavioral Data Analysis

Behavioral data, obtained only for 7/12 participants, were recorded and analyzed to verify that participants correctly performed the rhythmic tasks during the experiment. Inter-response-intervals (IRIs) and synchronization errors—the time lag between tap and beat onsets—were computed by averaging responses across all individual trials within each participant, and across all trials and all participants to generate group mean data.

### Magnetic Resonance Imaging Analysis

We used SPM99b (Wellcome Department of Cognitive Neurology) implemented in MATLAB (Mathworks Inc., Natick, MA) installed on UNIX workstations (SGI, Mountain View, CA) for initial data processing and subsequent statistical analysis. As described in detail below, the analysis strategy followed a two-stage process, first at the voxel-level for individual participants and then at the participant level to identify clusters having group-wise significant activation. We removed the first two functional MRI volumes from the entire run due to T1 overshoot effects; all remaining 328 volumes became entered into the data analysis. The functional MRI volumes from each participant were corrected for head movement that occurred during the functional MR data acquisition run, using the third scan as a reference, through a rigid body transformation using a least squares approach [44]. We used a template image based on average data provided by the Montreal Neurological Institute [45] and conformed to a standard coordinate referencing system [46]. Following image processing and resampling, the voxel size was 2 mm isotropic, and images were spatially smoothed using an isotropic gaussian kernel of 4 mm, full-width, half-maximum.

Images were analyzed using a two-stage approach. For the first analysis pass, the time series obtained from each participant was analyzed separately to identify activated voxels. The effects of the experimental design were estimated voxel-by-voxel [47]–[50]. The tasks were modeled as box-car functions and convolved with a synthetic hemodynamic response function. As noted, the first two volumes became discarded at the beginning of the run to account for equilibration of T1 effects, thereby leaving 53 volumes for the no-tap, 2 Hz listening task and 55 volumes for all remaining conditions for the subsequent analysis. The additional explanatory variables modeled in the statistical analysis included translations and rotations in the three axes of head movement obtained from the spatial registration to remove the components of the signal potentially correlated with head movement; a set of cosine basis functions to remove low-frequency confounds and a constant term. For each participant-specific model, linear compounds of the regression parameter estimates, that is, linear contrasts were used to estimate the size of the effects of interest. We first estimated significance at the voxel level by comparing the movement tasks with the no-movement condition that yielded effect size images for each participant for each of four stimulus and movement rate combinations, and then assessed differences between conditions across participants with one-way or paired t-tests. The two-stage analysis yielded a statistical parametric map of the t statistic for each effect of interest. We used a probability criterion of p≤0.001 at the voxel level and at p≤0.05 corrected for the number of sampled voxels at the cluster level, thereby correcting for multiple comparisons. At the cluster level (that reported in Results), we evaluated (1) movement vs. no movement for the isorhythmic and polyrhythmic paced movements; (2) the movement frequency effect (3 Hz vs. 2 Hz); (3) the stimulus frequency effect (3 Hz vs. 2 Hz), and (4) isorhythmic vs. polyrhythmic pacing, independent of stimulus or movement frequency. This last comparison could be considered the interaction between stimulus and movement frequency.

The statistical parametric maps were superimposed onto the standard brain supplied by SPM99, and localization of regions and Brodmann area (BA) assignments from these images were done using gyral and sulcal landmarks and coordinates provided by SPM99 and reference sources [46], [51]. BA assignments in the cerebral cortex were done using the senior author's (JNS) knowledge of neuroanatomy and by referring to the Talairach and Tournoux [46] atlas and other sources, while most gyral and sulcal nomenclature used a combination of Duvernoy [51] and Talairach and Tournoux [46]. Cerebellar nomenclature was derived from Schmahmann et al. [52]. In no cases, did we use the Talairach and Tournoux [46] atlas as the definitive source for activation cluster localization.

## Results

### Motor performance

**Figure 2:**
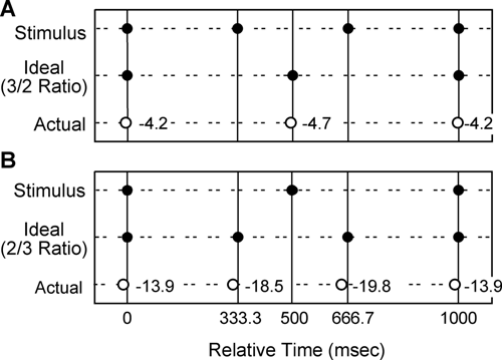
Behavioral results. Mean group performance of tapping (outside the MR environment) relative to the pacing cues in the 2/3 Hz (A) and the 3/2 Hz (B) tasks. Dots represent the relative occurrence of the auditory cue (upper row in each panel), the ideal relative time for occurrence of tapping (middle row), and the actual occurrence of tapping (group mean, lower panel), all across a 1 s interval. The numbers in the lower row indicate the lag/lead (msec) of the actual mean performance.

We measured the ability to perform the isorhythmic and the polyrhythmic, hemiola, movements in 7/12 participants separate from the MRI investigations; no behavioral measures were done during the functional MRI runs (see Materials and Methods and Discussion). Participants performed both the isorhythmically (not illustrated) and polyrhythmically (Fig. 2) paced movements with a high degree of accuracy consistent with prior data (see e.g., [7], [10], [12], [53]). Across the group, the mean (±SD) IRI for the isorhythmically paced 2 Hz movements was 499.7±31.3 ms and 333.2±27.5 ms for the isorhythmically paced 3 Hz movements; the observed performance did not deviate significantly from an expected mean rate of 2 Hz or 3 Hz (Wilcoxon signed rank test, p>0.19). The synchronization errors, that is, the time difference between onset of tap and onset of the beat, showed the well documented negative asynchrony of tapping slightly ahead of the beat [7]. The participants exhibited mean (±SEM) synchronization errors of −12.6±29.4 ms in the 2 Hz condition and −20.0±25.2 ms in the 3 Hz condition.

In the polyrhythmically conditions, mean IRIs were 498.5±36.6 ms for the 2 Hz movements paced by 3 Hz beeps and 330.7±28.5 ms for the 3 Hz movement, 2 Hz beeps, indicating a high degree of compliance (and performance success) in producing hemiola synchronization patterns. As will be noted, these IRIs had great similarity to comparable movement rates in the isorhythmic conditions. Further analysis of these data revealed a failure to reject the null hypothesis that the participants performed at the expected mean rate of 2 Hz or 3 Hz asynchronously to the driving stimulus (Wilcoxon signed rank test, p = 0.09, 3 Hz tapping; p = 1, 2 Hz tapping). Mean synchronization errors showed the expected negative asynchrony when aligned with the synchronous beat at the beginning of the period as well as when aligned with the mathematical subdivision of the period during the asynchronous tap-beat time relations during the rest of the period when no coincidence between beat and tap onsets was possible. For the condition with 2 Hz movement with 3 Hz beats, the synchronization error for the first movement was −4.2 ms and −4.7 ms for the second movement. For the condition with 3 Hz movements with 2 Hz beat, the synchronization error was −13.9 msec, −18.5 msec, and −19.8 ms for the first, second and third movements, respectively. The behavioral data of the examined participants showed conformity with predicted performance, lending credence to the conclusion that the rhythmic behavior was performed as requested during the MRI experiment.

Statistical comparisons of the mean IRIs and the means of the IRI standard deviations of the 2 Hz and 3 Hz movement conditions during isorhythmic and polyrhythmic tapping did not reach statistical significance (t-test, p≥0.05). During isorhythmic tapping, the synchronization errors between the 2 Hz and 3 Hz condition were also statistically non-significant (p≥0.05). The synchronization errors during asynchronous tapping were not compared statistically due to different stimulus alignments conditions across tapping movements (auditory beat present vs. mathematically inferred beat subdivisions).

### Brain activation

**Figure 3:**
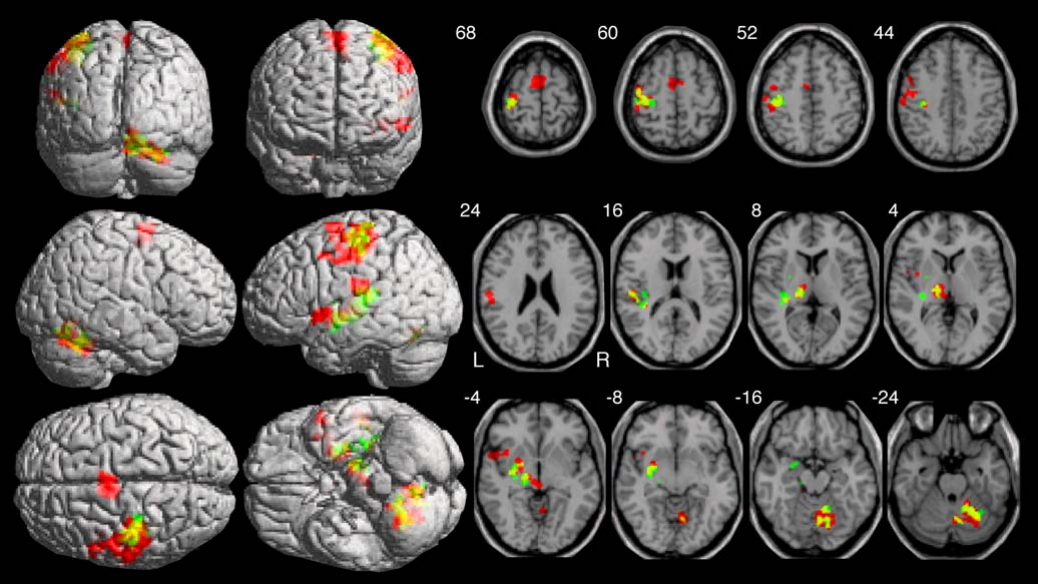
Brain activation during paced tapping. Foci of activation for isorhythmic-only tapping (green label), polyrhythmic-only tapping (red), and overlap (yellow) depicted on rendered projection images (left) and selected axial slice (right). Isorhythmic tapping activated M1, S1, and PMA, and the temporal operculum in contralateral neocortex as well as the basal ganglia and cerebellum. Polyrhythmic tapping activated the same structures, as well as the supramarginal gyrus, SMA, preSMA, cingulate cortex, and the middle and superior temporal gyri. Note greater extent of activation for polyrhythmic then isorhythmic tapping in commonly activated areas, and new areas of activation mostly for polyrhythmic tapping. Numbers next to upper left of each horizontal brain slice refer z-axis in MNI-Talairach space. Additional details in text; full reporting of the activated areas appears in Table 1.

When compared to no-movement, the tapping movements, collapsed across the iso- and polyrhythmically paced movement activated several structures including M1, PMA and SMA in the neocortex, the basal ganglia, and cerebellum (Fig. 3, yellow label, Table 1). As expected, the neocortical, basal ganglia and thalamic activation occurred primarily contralateral to the right-handed movement, whereas cerebellar activation occurred ipsilateral to the movement. Consistent with many previous observations (e.g., [54]), the midline frontal areas SMA and preSMA exhibited bilateral activation. While parietal cortex often becomes activated during simple, repetitive finger movements, movement-related activation commonly occurs more superiorly than the observed activation cluster that was located in the inferior portions of the contralateral supramarginal gyrus and extending into the superior temporal gyrus. When compared against no-movement and considered individually, the polyrhythmically paced movements (red and yellow label, Fig. 3, Table 1) yielded more overall activation than the isorhythmically paced movements (green and yellow label, Fig. 3, Table 1). The greater activation for polyrhythmically paced movements occurred contralateral to the hand movement in M1/S1, the thalamus, the putamen, and the parietal operculum and ipsilateral in the cerebellum. Contrary to many prior observations (e.g., [29], [55], but see [31]), no area exhibited differential activation relative to movement rate, though many of these studies used self-paced, and not stimulus-driven, repetitive movements. However, we found a frequency related effect for the stimulus-alone condition. That is, repeating the sounds at a higher rate without movement yielded more activation bilaterally in the anterior portions of the superior temporal gyrus that bordered on the opercular regions and in the right superior parietal lobule (Table 2, not illustrated).

**Table 1:** Activation related to polyrhythmic and isorhythmic movements compared to no movement.

		Polyrhythmic				Isorhythmic			
Cluster	Region (BA)	Coordinates			Extent	Coordinates			Extent
		x	y	z	(cc)	x	y	z	(cc)
L Fronto-parietal	M1 (4)	−32	−26	68	3.87	−28	−28	62	2.99
	S1 (1,2,3)	−48	−30	62		−44	−24	52	
	PMA (6)	−30	−20	66		−40	−20	62	
	GSM (40)	−48	−28	52		–	–	–	
L Fronto-parietal	S1 (1,2,3)	−58	−18	50	1.48	–	–	–	
	M1 (4)	−56	−12	40		–	–	–	
	PMAsup (6)	−42	−10	48		–	–	–	
	PMAinf (6)	−52	8	44		–	–	–	
Medial Frontal	L SMA(6)	−8	−8	68	2.06	–	–	–	
	R SMA (6)	2	−6	70		–	–	–	
	L preSMA (6)	−6	2	68		–	–	–	
	R preSMA (6)	2	2	68		–	–	–	
	L Cingulate (24)	−8	−4	56		–	–	–	
L Parietal-temporal	GSM/GST (40)	−50	−22	16	0.94	–	–	–	
	GSM (40)	−56	−26	24		–	–	–	
	GSM (40)	−56	−16	22		–	–	–	
L basal ganglia	Globus pallidus	−24	−8	−2	1.95	−34	−12	−6	1.26
	Putamen	−36	4	0		−28	−4	6	
L Insula	Insula	−52	10	−4	0.65	–	–	–	
L Insula	Insula	−32	−30	10	0.37	−32	−26	10	1.18
L temporal	Operculum (41)	–	–	–	–	−52	−22	16	0.3
L Thalamus	Ventral lateral	16	−24	8	2.11	−16	−28	0	1.38
	Medial	−6	−24	0		–	–	–	
R Cerebellum	Crus V	16	−48	−22	5.15	–	–	–	
	Crus VI	28	−62	−24		4	−66	−16	1.22
	Crus V	16	−50	−18	1.93	–	–	–	
	Crus VI	30	−62	−24		–	–	–	

**Table 2:** Activation related to frequency of movement.

		3 Hz>2 Hz			
Cluster	Region (BA)	Coordinates			Extent
		x	y	z	(cc)
R Parietal	SPL (7)	30	−42	56	0.18
R Temporal	GTS/Operculum (41)	44	−24	10	0.23
R Temporal	Operculum (41)	46	−16	−2	0.21
L Temporal	GTS/Operculum (41)	−20	−66	−18	0.20

**Figure 4:**
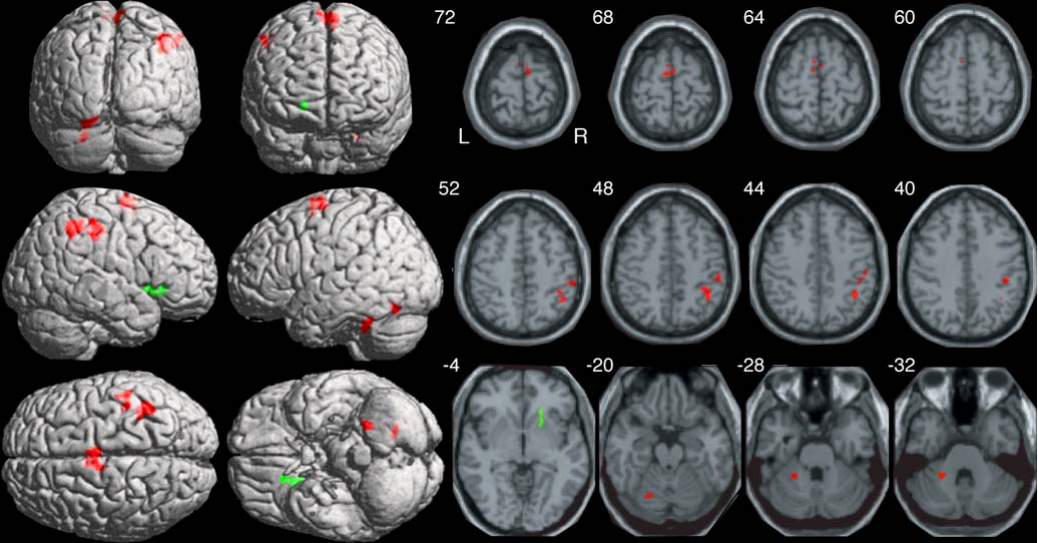
Hemiola specific activation. Brain regions exhibiting greater (red) or less (green) activation for polyrhythmic compared with isorhythmic tapping. Note greater extent of activation for polyrhythmic then isorhythmic tapping in ipsilateral inferior parietal lobule, contralateral cerebellum and bilateral SMA; the ipsilateral caudate-putamen exhibited decreased activation for the hemiola conditions. Other details as in Fig. 3.

The prior analysis demonstrated that two modes of auditory paced tapping— isorhythmic and polyrhythmic—yielded activation in many common structures, especially those commonly related to motor performance. However, these analyses did not reveal differential activation for two types of tapping tasks. Direct statistical comparison between functional MRI signals obtained during isorhythmically and polyrhythmically paced movements yielded a restricted brain network that exhibited differential activation between these two types of stimulus driven movements (Fig. 4, Table 3). Contrary to the sensory-motor network in contralateral neocortex, basal ganglia, and thalamus and ipsilateral cerebellum, the network with greater activation for polyrhythmic movements occurred ipsilateral to the movement in the telencephalon and contralateral in the cerebellum. In neocortex, SMA exhibited bilateral activation more for polyrhythmically paced movements, as did two separate clusters in the right supramarginal gyrus. Two additional small clusters in the left cerebellar hemisphere showed more activation for polyrhythmically paced than for isorhythmically paced movements. Finally, a cluster spanning the right anterior caudate and putamen yielded less activation for the asymmetrically paced movements in comparison to the symmetrically paced movements.

**Table 3:** Activation related to the polyrhythmic and isorhythmic pacing.

		Polyrhythmic>Isorhythmic				Isorhythmic>Polyrhythmic			
Cluster	Region (BA)	Coordinates			Extent	Coordinates			Extent
		x	y	z	(cc)	x	y	z	(cc)
Medial-frontal	L SMA (6)	−4	−6	70	0.58	–	–	–	
	R SMA (6)	4	−4	72		–	–	–	
R Parietal	GSM (40)	52	−32	50	0.51	–	–	–	
R Parietal	GSM (40)	38	−48	46	0.71	–	–	–	
R basal ganglia	Caudate nucleus	–	–	–	–	18	26	−4	0.38
	Putamen	–	–	–	–	18	14	−4	
L Cerebellum	Crus VI	−20	−66	−18	0.26	–	–	–	
L Cerebellum	Crus VI	−28	−42	−34	0.29	–	–	–	

## Discussion

The major finding reported here concerns activation patterns in motor-related brain regions during the direct comparison of polyrhythmic hemiola and isorhythmic timing patterns. We observed three types of activation patterns related to hemiola performance beyond that expected by performing repetitive finger movements. First, activation emerged during the hemiola condition bilaterally in SMA and ipsilaterally in the inferior parietal lobule. Second, a pattern of expanded or increased hemiola-related activation of structures exhibiting isorhythmic-related activation occurred in portions of contralateral cerebellar hemisphere. Third, a region encompassing portions of the putamen and nearby caudate nucleus exhibited decreased activation during hemiola rhythm production. These data suggest a differential sub-cortical role and an emergent neocortical role in mediating isorhythmic and hemiola rhythm production than that typically observed during movements.

The activation results do not seem related to motor performance changes, but more likely due to internal processing needed for integrating rhythmic auditory input with motor output. Indeed, the performance for the isorhythmic and polyrhythmic tasks exhibited substantial similarity in synchronization accuracy. The evident absence of motor performance differences for the two rhythm formation tasks suggests an integrative process that compensates for differences in input and internal processing demands to yield an equivalent motor output. Similar motor equivalence processes occur in many motor systems speech production and skeletal motor control [56], [57].

While we would have preferred to record motor performance in the MR environment—this technology had not yet implemented at the performance site when this experiment was conducted—prior work suggests that such data recording may not have necessity due to the selection of highly skilled musicians as participants. First, all participants had significant musical experience, and each had rigorous prescreening for the ability to perform the hemiola task. Second, while inexperienced individuals can perform polyrhythmic motor performance, as for example the asynchronous, hemiola structures, they do so only with difficultly [58]. Such difficulty can be overcome with practice and learning (e.g., [58], [59]), as for example, with musical training. Polyrhythmic patterns have commonality in music performance and composition [16]. Therefore, the semi-professional and professional musicians included in this work must develop capability to generate stable hemiola patterns similar to simple isochronous rhythms or rhythmic patterns involving harmonic relationships, such as 1∶2 or 1∶3 movement [32], [60]. The temporal and motor strategies of polyrhythmic performance in musicians and non-musicians have been studied extensively [42]. Thus, polyrhythmic performance in a musical context can be considered a categorical skill independent from environmental context [61]. In conclusion, it appears that performing hemiola structures with high accuracy is a categorical skill; either one can perform hemiola structures or one cannot; one does not forget and need to relearn hemiola structures; and those who can perform such structures do so reliably and reproducibly even in the face of distracting events (such as 100 other instrument sounds in a symphonic performance). Therefore, the inability to measure movement performance during the functional MRI acquisitions does not seem to pose a serious limitation for the interpretation of the obtained results.

The current brain activation findings may suggest that neural processes that generate equivalent motor outputs use the same network of brain areas. Indeed, others have shown that activation in a neocortical-subcortical network may reflect the behavioral aspects of motor equivalence [62], [63]. Kelso et al. [62] found a task-independent signature of movement velocity in regions encompassing the precentral gyrus during syncopated and synchronized rhythmic movements, though they did not localize the source of the activation. In another study, more aligned with those investigating motor equivalence, Rijntjes et al. [63] found an overlapping network for signing ones name with the toes and the finger. This “signature” network encompassed structures bilaterally in PMA, SMA, areas flanking the intraparietal sulcus, thalamus, and cerebellum. While our data have some similarity to those of Kelso et al. [62] and Rijntjes et al. [63], we found a specific unilateral network that distinguished repetitive movements paced asynchronously or synchronously, and this network had a laterality opposite from that expected by simple motor performance. Thus, the current results do not provide explicit support for brain substrates of motor equivalence, at least for simple repetitive movements paced by auditory cues. Since we found different activation patterns for the hemiola and isorhythmic patterning, even when movement frequency was matched, the findings of Kelso et al [62] do not seem to generalize to other tasks that yield that same output (e.g., 3 Hz tapping driven by 3 Hz or 2 Hz sounds). Instead, new patterns of activation emerged due to novel sensory-motor patterns.

Two potential alternate explanations for the activation differences between the polyrhythmic and isorhythmic movements relate to differences in difficulty and attentional demands between the two tasks. Performing the polyrhythmic hemiola structures clearly requires more effort than performing on-the-beat rhythms, as indicated by the somewhat poorer accuracy and variability the occurred during this task. Indeed, we needed to recruit participants with significant prior experience with musical instrument training, since non-musicians had little ability to perform a hemiola rhythm. Similarly, greater demand upon attention resources likely occurs when performing the hemiola rhythms. The brain imaging results suggest that neither difficulty nor attention yielded the observed activation pattern. We found no evidence of activation in structures commonly associated with task difficulty—such as the anterior cingulate cortex and portions of prefrontal cortex [64]—or attention to sensory events [65] or movement [54], [66]–[69]. Spatial attention right parietal regions activated during the polyrhythmic tasks may have relation to spatial attention [66], [69]. While both rhythm formation tasks activated left, but not right, parietal areas when compared to no-movement, the polyrhythmic condition task yielded greater activation in the right parietal structures, perhaps indicating a greater need to sensory-motor spatial integration for producing polyrhythms; Vuust et al. [23] have observed activation in a nearby region of the right parietal lobe when participants tapped to a main meter while listening to a counter meter. Neural responses in some visual processing areas can exhibit attention influences (e.g., [70], [71]), suggesting that the brain areas exhibiting more activation for performing the hemiola structure might have related to attention demands. However, Astafiev et al. [72] found that attention and preparing pointing movements largely activate fundamentally different parietal and frontal cortical networks. Considering these findings, it seems unlikely that alternative explanations of difficulty and attention accounted for the current results.

Several brain regions appear to have involvement in elaborating timing behavior, including the neocerebellum [30], [73]–[75], basal ganglia [2], prefrontal cortex [76], [77], and parietal lobe [23], though how each contributes to rhythm formation and motor timing remains somewhat unclear. Increasing temporal complexity in timing tasks increases activation in the SMA and cerebellum [78], a finding that we have confirmed. Basal ganglia activation during both rhythm conditions compared to no movement was differentially stronger during isorhythmic than polyrhythmic hemiola tapping whereas cerebellar activation was stronger during the hemiola task. This differential dissociation in activation patterns between the two systems may support an important role of the cerebellum in neural computations related to rhythmic complexity (also see [75]). However, several studies show auditory rhythmic entrainment of motor functions occurs in patients with cerebellar and basal ganglia disorders [74], [79], [80]. Therefore, during auditory rhythmic synchronization, neocortical as well as basal ganglia and cerebellar circuits may become involved in differential aspects of processing timing information. This information, already coded in precise neural excitation patterns within auditory pathways could be projected into relevant motor areas in a highly distributed fashion via oscillatory neural resonance mechanisms [22], [81]–[84]. Further support for a specific cerebellar role in timing mechanisms comes from Rao et al. [2], who found late recruitment of cerebellar networks, after prior basal ganglia activation, in a temporal processing task and from Stephan et al. [85] who found ipsilateral cerebellar activation during motor synchronization to auditory rhythm regardless of rhythmic condition whereas posterior bilateral activations developed during rhythmic modulations of increasing magnitude. Taken together, we may postulate that basal ganglia involvement is related to basic timing and sequencing aspects of rhythmic motor performance whereas cerebellar activations serve sensory-motor integrative optimization functions that operate particularly as essential requirements in tasks with high degrees of temporal complexity. The actual clock timing (duration coding) of the motor performance may simply be due to the entrainment of neural motor codes by the precise neural excitation patterns in the auditory pathway induced by auditory rhythm.

Lastly, the results of this study hold considerable interest for the neural basis of musical rhythm formation. Since periodic perceptual grouping—based on matching sound events to pulse driven template structures—represents the most appropriate basis for a definition of musical rhythm, rhythmic synchronization tasks would be one of the most meaningful and realistic experimental paradigms to approach the study of the neural correlates of rhythm. They would most closely reflect the process of intrinsic temporal pattern formation within a synchronized pulse structure as the core effort in the perception and production of musical rhythm [85]. Study designs that employ the production or recognition of sequences of various time intervals without implicitly felt pulse structure [86] or that rely on discrimination tasks based on working memory would seem less central to musical rhythm since they rely on shared processes with other forms of non-rhythmic time processing such as that found in speech processing, working memory, and other functions.

Furthermore, the performance of rhythmic hemiolas used in our study is highly unique to rhythm in music and represents one of the most difficult tasks in musical rhythm performance. Previous evidence has shown that serial groupings like a 2∶3 polyrhythm, when performed in bimanual tapping tasks, usually become organized in an integrated temporal structure rather than a segregated streamed percept [58], [60]. Integrated organization may be indicative, however, of performing a chained performance of successive tap events of various durations rather than a true alignment of separate rhythmic pattern streams into a common pulse structure as required in the tasks performed here [58]. This phenomenon is easily observed in musically naive people who can learn quickly to tap out hemiolas once they determine how the succession of taps sounds or feels without any perception of the polyrhythmic streaming of two distinct rhythmic patterns. This lack of perception becomes obvious when (unsuccessfully) attempting to tap hemiolas against a given isorhythmic background pulse, thus having to generate periodic groupings within a pulse template structure. Thus, the experimental hemiola task in our study (motor response aligned with rhythmic sensory cues) represents a ‘realistic’ simulation of one of the most difficult tasks in musical rhythm performance. The neural networks associated with both the isorhythmic and the polyrhythmic hemiola conditions in our study can therefore—with a good degree of confidence—be considered core components of the neurobiological circuitry subserving rhythm formation in music.

## References

[1] Ivry RB, Keele SW (1989). Timing functions of the cerebellum.. J Cogn Neurosci.

[2] Rao SM, Mayer AR, Harrington DL (2001). The evolution of brain activation during temporal processing.. Nat Neurosci.

[3] Lewis P, Wing A, Pope P, Praamstra P, Miall R (2004). Brain activity correlates differentially with increasing temporal complexity of rhythms during initialisation, synchronisation, and continuation phases of paced finger tapping.. Neuropsychologia.

[4] Spencer RM, Verstynen T, Brett M, Ivry R (2007). Cerebellar activation during discrete and not continuous timed movements: an fMRI study.. Neuroimage.

[5] Kelso JAS, Delcolle JD, Schoener G, Jeannerod M (1990). Action-perception as a pattern formation process.. Attention and performance XIII.

[6] Mates J (1994). A model of synchronization of motor acts to a stimulus response. I. Timing and error corrections.. Biol Cybern.

[7] Aschersleben G, Prinz W (1995). Synchronizing actions with events: The role of sensory information.. Percept Psychophys.

[8] Vorberg D, Wing A, Heuer H, Keele SW (1996). Modeling variability and dependence in timing.. Handbook of perception and action, volume 2.

[9] Chen R, Gerloff C, Hallett M, Cohen LG (1997). Involvement of the ipsilateral motor cortex in finger movements of different complexities.. Ann Neurol.

[10] Thaut MH, Miller RA, Schauer ML (1998). Multiple synchronization strategies in rhythmic sensorimotor tasks: Phase vs. period corrections.. Biol Cybern.

[11] Thaut MH, Stephan KM, Wunderlich G, Schicks W, Tellmann L (2008). Distinct cortico-cerebellar activations in rhythmic auditory motor synchronization.. Cortex, in press..

[12] Mueller K, Schmitz F, Scnitzler A, Freund HJ, Aschersleben G (2000). Neuromagnetic correlates of sensorimotor synchronization.. J Cogn Neurosci.

[13] Roberts S, Eyckholt R, Thaut MH (2000). Search for correlations and evidence for deterministic chaos in rhythmic motor control of the human brain.. Phys Rev E.

[14] Repp BH (2001). Processes underlying adaptation to tempo changes in sensorimotor synchronization.. Hum Mov Sci.

[15] Stephan KM, Thaut MH, Wunderlich G, Schicks W, Tian B (2002). Conscious and subconscious sensorimotor synchronization: Prefrontal cortex and the influence of awareness.. Neuroimage.

[16] Parncutt R (1994). A perceptual model of pulse salient and metrical accent in musical rhythm.. Music Percept.

[17] Snyder B (2000). Music and memory..

[18] Palmer C, Krumhansl CL (1990). Mental representations for musical meter.. J Exp Psychol Hum Percept Perform.

[19] Epstein D (1985). Tempo-relations: a cross-cultural study.. Music Theory Spectrum.

[20] Cariani PA, Delgutte B (1996). Neural correlates of the pitch of complex tones. I. Pitch and pitch salience.. J Neurophysiol.

[21] Cariani PA, Delgutte B (1996). Neural correlates of the pitch of complex tones. II. Pitch shift, pitch ambiguity, phase invariance, pitch circularity, rate pitch, and the dominance region for pitch.. J Neurophysiol.

[22] Tecchio F, Salustri C, Thaut MH, Pasqualetti P, Rossini PM (2000). Conscious and preconscious adaptation to rhythmic stimuli: A magnetoencephalographic study of human brain responses.. Exp Brain Res.

[23] Vuust P, Roepstorff A, Wallentin M, Mouridsen K, Ostergaard L (2006). It don't mean a thing. Keeping the rhythm during polyrhythmic tension, activates language areas (BA47).. Neuroimage.

[24] Platel H, Price C, Buron JC, Wise R, Lambert J (1997). The structural components of music perception.. Brain.

[25] Harrington DL, Haaland KY, Knight RT (1998). Cortical networks underlying mechanisms of time perception.. J Neurosci.

[26] Parsons L, Thaut M (2001). Functional neuroanatomy of the perception of musical rhythm in musicians and nonmusicians.. Neuroimage.

[27] Halsband U, Ito N, Tanji J, Freund HJ (1993). The role of premotor cortex and the supplementary motor area in the temporal control of movement in man.. Brain.

[28] Boecker H, Kleinschmidt A, Requardt M, Haenicke W, Merboldt KD (1994). Functional cooperativity of human cortical motor control areas during self-paced simple finger movements: A high resolution MRI study.. Brain.

[29] Schlaug G, Sanes JN, Thangaraj V, Darby DG, Jancke L (1996). Cerebral activation covaries with movement rate.. Neurorep.

[30] Penhune VB, Zatorre RJ, Evans A (1998). Cerebellar contributions to motor timing: A PET study of auditory and visual rhythm reproduction.. J Cogn Neurosci.

[31] Kim JA, Eliassen JC, Sanes JN (2005). Movement quantity and frequency coding in human motor areas, J Neurophysiol.

[32] Ullen F, Forssberg H, Ehrsson HH (2003). Neural networks for the coordination of the hands in time.. J Neurophysiol.

[33] Harrington DL, Haaland KY, Hermanowicz N (1998). Temporal processing in the basal ganglia.. Neuropsychology.

[34] Spencer RM, Ivry RB (2005). Comparison of patients with Parkinson's disease or cerebellar lesions in the production of periodic movements involving event-based or emergent timing.. Brain Cogn.

[35] Cooper G, Meyer LB (1960). The rhythmic structure of music..

[36] Pressing J (2002). Black Atlantic rhythm: Its computational and transcultural foundations.. Music Percep.

[37] Blacking J (1973). How musical is man..

[38] Deutsch D (1983). The generation of two isochronous sequences in parallel.. Percep Psychophys.

[39] Summers J (2002). Practice and training in bimanual coordination tasks.. Brain Cogn.

[40] Bogacz S (2005). Understanding how speed affects performance of polyrhythms.. J Mot Behav.

[41] Oldfield RC (1971). The assessment and analysis of handedness: the Edinburgh inventory.. Neuropsychologia.

[42] Krampe RT, Kliegl R, Mayr U, Engbert R, Vorberg D (2000). The fast and the slow of skilled bimanual rhythm production: parallel versus integrated timing.. J Exp Psychol: Hum Percept Perform.

[43] Kwong KK, Belliveau JW, Chesler DA, Goldberg IE, Weisskoff RM (1992). Dynamic magnetic resonance imaging of human brain activity during primary sensory stimulation.. Proc Natl Acad Sci U S A.

[44] Friston KJ, Ashburner J, Poline JB, Frith CD, Heather JD (1995). Spatial registration and normalization of images.. Hum Brain Mapp.

[45] Mazziotta JC, Toga AW, Evans A, Fox P, Lancaster J (1995). A probabilistic atlas of the human brain: theory and rationale for its development. The international consortium for brain mapping (ICBM).. Neuroimage.

[46] Talairach J, Tournoux P (1988). Co-planar stereotaxic atlas of the human brain: 3-dimensional proportional system: an approach to cerebral imaging..

[47] Friston KJ, Frith CD, Liddle PF, Frackowiak RS (1991). Comparing functional (PET) images: the assessment of significant change.. J Cereb Blood Flow Metab.

[48] Friston KJ, Worsley KJ, Frackowiak RSJ, Mazziotta JC, Evans AC (1994). Assessing the significance of focal activations using their spatial extent.. Human Brain Mapp.

[49] Friston KJ, Holmes AP, Worsley KJ (1999). How many subjects constitute a study?. Neuroimage.

[50] Worsley KJ, Evans AC, Marrett S, Neelin P (1992). A three-dimensional statistical analysis for CBF activation studies in human brain.. J Cereb Blood Flow Metab.

[51] Duvernoy HM (1991). The human brain: Surface three dimensional sectional anatomy and MRI..

[52] Schmahmann JD, Doyon J, McDonald D, Holmes C, Lavoie K (1999). Three-dimensional MRI atlas of the human cerebellum in proportional stereotaxic space.. Neuroimage.

[53] Thaut M, Kenyon GP (2003). Rapid motor adaptations to subliminal frequency shifts during syncopated rhythmic sensorimotor synchronization.. Hum Mov Sci.

[54] Indovina I, Sanes JN (2001). Combined visual attention and finger movement effects on human brain representations.. Exp Brain Res.

[55] Jancke L, Specht K, Mirzazade S, Loose R, Himmelbach M (1998). A parametric analysis of the ‘rate effect’ in the sensorimotor cortex: a functional magnetic resonance imaging analysis in human subjects.. Neurosci Lett.

[56] Lashley K (1930). Basic neural mechanisms in behavior.. Psychol Rev.

[57] Gracco VL, Abbs JH (1988). Central patterning of speech movements.. Exp Brain Res.

[58] Klapp ST, Nelson JM, Jagacinski RJ (1998). Can people tap concurrent bimanual rhythms independently.. J Motor Behav.

[59] Tajima M, Choshi K (1999). Pattern formation in polyrhythmic tapping at a self-paced tempo.. Percept Mot Skills.

[60] Jagacinski RJ, Marshburn E, Klapp ST, Jones MR (1988). Tests of parallel versus integrated structure in polyrhythmic tapping.. J Motor Behav.

[61] Beauvillain C, Fraisse P (1984). On the temporal control of polyrhythmic performance Music Percept.

[62] Kelso JA, Fuchs A, Lancaster R, Holroyd T, Cheyne D (1998). Dynamic cortical activity in the human brain reveals motor equivalence.. Nature.

[63] Rijntjes M, Dettmers C, Büchel C, Kiebel S, Frackowiak RS (1999). A blueprint for movement: functional and anatomical representations in the human motor system.. J Neurosci.

[64] Barch DM, Braver TS, Nystrom LE, Forman SD, Noll DC (1997). Dissociating working memory from task difficulty in human prefrontal cortex.. Neuropsychologia.

[65] Corbetta M, Shulman GL (2002). Control of goal-directed and stimulus-driven attention in the brain.. Nat Rev Neurosci.

[66] Rushworth MF, Ellison A, Walsh V (2001). Complementary localization and lateralization of orienting and motor attention.. Nat Neurosci.

[67] Rushworth MF, Krams M, Passingham RE (2001). The attentional role of the left parietal cortex: the distinct lateralization and localization of motor attention in the human brain.. J Cogn Neurosci.

[68] Rushworth MF, Paus T, Sipila PK (2001). Attention systems and the organization of the human parietal cortex.. J Neurosci.

[69] Rounis E, Yarrow K, Rothwell J (2007). Effects of rTMS conditioning over the fronto-parietal network on motor versus visual attention.. J Cogn Neurosci.

[70] Treue S, Maunsell JH (1996). Attentional modulation of visual motion processing in cortical areas MT and MST.. Nature.

[71] McAdams CJ, Maunsell JH (2000). Attention to both space and feature modulates neuronal responses in macaque area V4.. J Neurophysiol.

[72] Astafiev SV, Shulman GL, Stanley CM, Snyder AZ, Van Essen DC (2003). Functional organization of human intraparietal and frontal cortex for attending, looking, and pointing.. J Neurosci.

[73] Spencer RM, Zelaznik HN, Diedrichsen J, Ivry RB (2003). Disrupted timing of discontinuous but not continuous movements by cerebellar lesions.. Science.

[74] Molinari M, Leggio MH, Cerasa A, Thaut MH (2005). Sensorimotor transduction of time information is preserved in subjects with cerebellar damage.. Brain Res Bull.

[75] Del Olmo MF, Cheeran B, Koch G, Rothwell JC (2007). Role of the cerebellum in externally paced rhythmic finger movements.. J Neurophysiol.

[76] Mangels JA, Ivry RB, Shimizu N (1998). Dissociable contributions of the prefrontal and neocerebellar cortex to time perception.. Brain Res Cogn Brain Res.

[77] Macar F, Lejeune H, Bonnet M, Ferrara A, Pouthas V (2002). Activation of the supplementary motor area and of attentional networks during temporal processing.. Exp Brain Res.

[78] Janata P, Grafton ST (2003). Swinging in the brain: shared neural substrates for behavior related to sequencing and music.. Nat Neurosci.

[79] O'Boyle DJ, Freeman JS, Cody FWJ (1996). The accuracy and precision of timing of self-paced, repetitive movements in subjects with Parkinson's disease.. Brain.

[80] Thaut MH, Kenyon GP, Schauer ML, McIntosh GC (1999). The connection between rhythmicity and brain function.. IEEE Eng Med Biol.

[81] Buonomano DV, Merzenich MM (1995). Temporal information transformed into a spatial code by a neural network with realistic properties.. Science.

[82] Buonomano DV (2000). Decoding temporal information: a model based on short-term synaptic plasticity.. J Neurosci.

[83] Durstewitz D (2003). Self-organizing neural integrator predicts interval times through climbing activity.. J Neurosci.

[84] Kimpo RR, Theunissen FR, Doupe AJ (2003). Propagation of correlated activity through multiple stages of a neural circuit.. J Neurosci.

[85] Stephan KM, Thaut MH, Wunderlich G, Schicks W, Tian B (2002). Cortico-cerebellar circuits and temporal adjustments of motor behavior..

[86] Jackendoff R, Lehrdahl F, Clynes M (1982). A grammatical parallel between music and language.. Music, mind and brain.

[87] Povel DJ, Essens P (1985). Perception of temporal patterns.. Music Percept.

